# 579 Incidence of Acute Kidney Injury with Hydroxocobalamin Administration

**DOI:** 10.1093/jbcr/iraf019.208

**Published:** 2025-04-01

**Authors:** Adalah Yahia, Amima Mahmood, Jacob Pifer, Michael White

**Affiliations:** Detroit Receiving Hospital Burn Center; Detroit Receiving Hospital; Henry Ford Health; Michael & Marian Ilitch Department of Surgery, Wayne State University School of Medicine, Detroit Medical Center

## Abstract

**Introduction:**

Hydroxocobalamin is frequently administered to patients with inhalation injuries. Recent literature has raised concerns about its potential association with an increased risk of acute kidney injury (AKI). This study aims to investigate the incidence of AKI following hydroxocobalamin administration in this population.

**Methods:**

This retrospective cohort study examined 10 years of data from a single adult Burn Center, including patients with confirmed inhalation injuries and/or those treated with hydroxocobalamin between January 1, 2013, and January 1, 2023. The primary outcome was the incidence of AKI, classified using KDIGO criteria, within 7 days of hospital admission. Secondary outcomes included the incidence and timing of renal replacement therapy, 28-day all-cause mortality, and lactate clearance up to 72 hours post-admission. In the hydroxocobalamin group, safety endpoints included the incidence and severity of AKI within 7 days of administration and the cause of AKI.

**Results:**

A total of 312 patients met inclusion criteria, with 167 in the hydroxocobalamin group and 145 in the control group. Baseline characteristics were not matched, with the hydroxocobalamin group being more critically ill and the control group having more chronic illness. There was no statistically significant difference in the incidence of AKI within 7 days of hospital admission between those who received hydroxocobalamin and those who did not (38.9% vs. 32.4%, p=0.345). Secondary outcomes showed a lower 28-day mortality rate in the control group (7.1% vs. 18.9%, p=0.029). Lactate clearance was also significantly higher in the control group (median 2.09, IQR 1.29-2.6) compared to the hydroxocobalamin group (median 3.05, IQR 1.56-5.72, p=0.00056), though patients in the hydroxocobalamin group had higher initial lactate levels within the first 24 hours. The incidence of renal replacement therapy for AKI was higher in the hydroxocobalamin group compared to controls (21.4% vs. 6.3%, p=0.071).

**Conclusions:**

This study did not find a statistically significant association between hydroxocobalamin use and AKI in patients with inhalation injuries. However, several limitations were noted, including the lack of matched baseline characteristics, as critically ill patients were more likely to receive hydroxocobalamin. Further prospective research is warranted to determine whether hydroxocobalamin contributes to AKI, the extent of AKI (e.g., requiring renal replacement therapy), and to clarify potential risks and benefits when groups are appropriately matched.

**Applicability of Research to Practice:**

Despite concerns in the literature about the potential risk of AKI with hydroxocobalamin, this large retrospective study did not find a significant increase in AKI incidence associated with its use. However, further prospective studies are needed to definitively assess its impact on renal function and to explore the potential clinical benefits and risks.

**Funding for the Study:**

N/A

